# The meconium microbiota shares more features with the amniotic fluid microbiota than the maternal fecal and vaginal microbiota

**DOI:** 10.1080/19490976.2020.1794266

**Published:** 2020-08-02

**Authors:** Qiuwen He, Lai-Yu Kwok, Xiaoxia Xi, Zhi Zhong, Teng Ma, Haiyan Xu, Haixia Meng, Fangqing Zhao, Heping Zhang

**Affiliations:** a Key Laboratory of Dairy Biotechnology and Engineering, Ministry of Education P.R.C, Inner Mongolia Agricultural University, Huhhot, China; b Key Laboratory of Dairy Products Processing, Ministry of Agriculture and Rural Affairs P.R.C., Inner Mongolia Agricultural University, Huhhot, China; c Department of Gynecology and Obstetrics, Affiliated Hospital of Inner Mongolia Medical University, Huhhot, China; d Computational Genomics Lab, Beijing Institutes of Life Science, Chinese Academy of Sciences, Beijing, China

**Keywords:** Meconium, amniotic fluid, vaginal fluid, *in utero* colonization, sterile womb paradigm, maternal-neonate transmission

## Abstract

The early-life gut microbiota is associated with potential development of diseases in adulthood. The sterile womb paradigm has been challenged by recent reports that revealed the presence of the meconium, amniotic fluid, and placenta microbiome. This study aimed to explore the maternal origin of the microbiota of neonate meconium by using the PacBio single-molecule real-time circular consensus sequencing technology. Such technology could produce high fidelity reads of full-length 16S rRNA genes, improving the sensitivity and specificity of taxonomic profiling. It also reduced the risk of false positives. This study analyzed the full-length 16S rRNA-based microbiota of maternal samples (amniotic fluid, feces, vaginal fluid, saliva) and first-pass meconium of 39 maternal-neonate pairs. Alpha- and beta-diversity analyses revealed sample type-specific microbiota features. Most sample types were dominated by sequences representing different genera (*Lactobacillus* and *Curvibacter* in the amniotic fluid and vaginal fluid microbiota; *Bacillus* and *Escherichia*/*Shigella* in the meconium microbiota; *Bacteroides* and *Faecalibacterium* in the maternal fecal microbiota; *Streptococcus* and *Prevotella* in the maternal saliva microbiota). Moreover, specific operational taxonomic units (OTUs) were identified in all sample types. Dyad analysis revealed common OTUs between the meconium microbiota and microbiota of multiple maternal samples. The meconium microbiota shared more features with the amniotic fluid microbiota than the maternal fecal and vaginal microbiota. Our results strongly suggested that the meconium microbiota was seeded from multiple maternal body sites, and the amniotic fluid microbiota contributed most to the seeding of the meconium microbiota among the investigated maternal body sites.

## Introduction

The gut microbiota is closely connected with human health. Dysbiotic shifts in the gut microbiota are associated with various diseases, including obesity, inflammatory bowel disorder, autoimmune diseases, and gastrointestinal cancer.^1^ Stewart et al. reported that the gut microbiome development progressed through three distinct phases: a developmental phase, a transitional phase, and a stable phase; and breast milk feeding was the most significant factor influencing the microbiome structure and development.^2^ The gut microbiota in early life changed through interacting with the developing intestinal immune system.^3^ Defects in the development of invariant natural killer T cells and B cells in germ-free mice could be partly corrected by early post-natal colonization of commensal bacteria,^4,5^ suggesting that age-sensitive exposure to the normal microbial flora provided critical developmental cues for the immune system. Moreover, colonizing pregnant mice reprogrammed early postnatal innate immune development by increasing the expression of genes encoding epithelial antibacterial peptides and metabolic pathways relevant to microbial molecules.^6^ Thus, an in-depth understanding of the role of maternal bacteria in the seeding of the infant gut microbiota would help design early intervention strategies to improve human health, particularly in the aspect of immune system development. However, the origin of the neonatal gut microbiota remains controversial.^7^

Recently, the sterile womb paradigm has been challenged. Jiménez et al. isolated bacteria from umbilical cord blood of healthy neonates and from murine amniotic fluid, suggesting that term fetuses were not completely sterile and that a prenatal mother-to-child efflux of commensal bacteria might exist.^8^ The highly similar nucleotide variation patterns in some gut microbes of maternal-neonate pairs suggested potential vertical transmission of certain bacterial strains from mother to child.^9^ Gestational diabetes mellitus was associated with concordant alterations of maternal and neonatal microbiota.^10^ A strain-resolved metagenomic analysis revealed that the infant microbiome was largely acquired and transmitted maternally.^11^ The *in utero* colonization hypothesis was further supported by the detection of microbes and/or microbiomes in samples of meconium,^12–14^ amniotic fluid,^15,16^ placenta,^17–22^ and uterus^23,24^ by metagenomic sequencing, conventional microbial cultivation and/or microscopic methods. We thus hypothesized the existence of a maternally derived neonatal microbiome.

Although a large body of studies has reported the existence of microbiomes of the placenta, amniotic fluid, the chorionic and amniotic membranes in normal, uncomplicated pregnancies, several studies presented contrary findings. Rehbinder et al. analyzed the amniotic fluid microbiota of 24 mothers and concluded that amniotic ﬂuid was sterile in uncomplicated term pregnancy until uterine contractions and/or rupture of membranes.^25^ Lim et al. found no obvious difference between the microbiota of amniotic fluid obtained from uncomplicated term pregnancies and buffer negative controls,^26^ and de Goffau et al. found no evidence to support the existence of a placental microbiome in both complicated and uncomplicated pregnancies.^27^ These studies supported the sterile womb paradigm and disproved the *in utero* colonization hypothesis. They further argued that the presence of a meconium microbiota in previous studies was merely the results of DNA contamination originated from laboratory reagents or acquired during delivery.

The accuracy of profiling microbial communities in low microbial biomass samples (e.g., placenta, amniotic fluid, meconium) has been hindered by our ability to distinguish the authentic signals beyond the level of background contamination. The placental microbiome was firstly characterized metagenomically by Aagaard et al. (2014) using whole-genome shotgun sequencing and 454 pyrosequencing technologies.^17^ The 454 pyrosequencing technology produced sequences of medium read length (~450 bp).^28^ By analyzing samples collected from multiple human body sites of pregnant and nonpregnant subjects, Aagaard et al. found that the placental microbiome comprised nonpathogenic commensals most akin to the oral microbiome.^17^ In contrast, de Goffau et al. (2019) failed to identify distinguishable signals between the placental samples and contaminant controls by sequencing relatively short reads covering the V1-V2 hypervariable regions of the 16S rRNA (~260 bp).^27^ The conflicting inferences could be resulted from the chosen technologies that relied on different sub-regions and read lengths of 16S rRNA genes, resulting in different power of taxonomic resolution. Moreover, the long-read sequencing technology was potentially advantageous in reducing the contamination risk for metagenomic profiling of low microbial biomass samples, and employing thoughtful filtering settings and vigorous contaminant controls might further ‘decontaminate’ the putative contaminant amplicon sequence variants.^22^

Stinson et al. applied the PacBio single-molecule real-time (SMRT) sequencing technology together with workflows aiming to reduce contamination, showing the existence of the meconium and amniotic fluid microbiome beyond the level of background contamination.^16^ The PacBio SMRT sequencing technology has the major advantage of producing long reads. It has been used to profile the microbial communities of various samples based on sequencing the full-length 16S rRNA gene to provide more precise species and strain-level data.^29,30^ Such fine-scale taxonomic resolution was unachievable by other currently available sequencing platforms. The high random error rate of PacBio sequencing could be rectified by the circular consensus sequencing (CCS) strategy, which created high fidelity reads by multiple passes of template molecules, achieving a low error rate of 0.007%.^31^ Thus, combining these methods would enable more accurate taxonomic and phylogenetic resolution of microbial communities. Moreover, different sequences assigned under the same operational taxonomic unit (OTU) were closely related phylotypes, allowing OTU tracking analysis.

This study analyzed the high taxonomic resolution microbiota profiles of samples of 39 maternal-neonate pairs (Table S1) to investigate the contribution of the microbiota of the maternal feces (MF), vaginal fluid (MV), amniotic fluid (AF), and saliva (MS) to the seeding of the meconium microbiota (IF) by using the PacBio SMRT sequencing in combination with the CCS strategy.

## Results

### Dataset features and alpha-diversity

A total of 1,118,229 of 16S rRNA raw reads were generated from the 192 samples (mean = 5,824; range = 1,257–22,214; SD = 2,891). A total of 858,981 reads were delimited through PyNAST alignment and 100% sequence identity clustering. A total of 241,042 different OTUs (1,255 different OTUs/sample) were identified at 98.65% sequence similarity.

The Shannon diversity curves leveled off, suggesting that the sequencing depth was enough to capture a representative microbial diversity (Fig. S1a). The values of Shannon diversity indexes varied greatly between groups (meconium, 6.92 ± 0.75; amniotic fluid, 4.68 ± 1.94; maternal feces, 7.77 ± 0.91; vaginal fluid; 3.80 ± 1.75; maternal saliva, 8.11 ± 0.54). Pairwise comparison by Mann–Whitney test on Shannon diversity indexes revealed significant differences between most sample groups (*P < *.001 in most cases, except between amniotic fluid and vaginal fluid, *P < *.05; between maternal feces and saliva, *P* = .09; Fig. S1b). The coverage depth ranking was shown by the rank–abundance curves; a longer OTU ‘tail’ represented higher microbial diversity and richness. The maternal feces samples had apparently longer ‘tail’, whereas the vaginal fluid samples had the shortest ‘tail’ (Fig. S1 c).

### Microbiota composition of five sample groups

The microbiota composition of all samples was presented at different taxonomic levels (Fig. S2).

### Beta-diversity of the microbiota of the meconium and maternal samples

**Figure 1.**
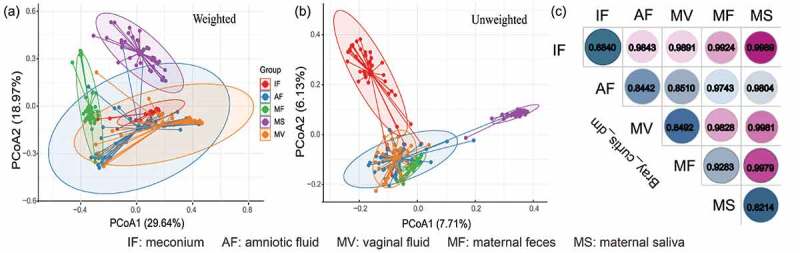
Dissimilarity-based multivariate analyses of microbiota communities of different sample types. Score plots of principal coordinates analysis (PCoA) of gut microbiota communities based on (a) weighted and (b) unweighted UniFrac distances. (c) Bray-Curtis dissimilarity matrix calculated based on microbial abundance patterns of operational taxonomic units (OTUs) of different sample types. A smaller value of the dissimilarity index indicates a higher similarity (i.e., not dissimilar) between samples. IF: meconium; AF: amniotic fluid; MF: maternal feces; MS: maternal saliva; MV: vaginal fluid.

The difference in the microbiota structure between the meconium and other maternal samples was evaluated by principal coordinate analysis (PCoA) and Bray–Curtis dissimilarity (Figure 1). Symbols representing maternal saliva clustered distinctly on the weighted and unweighted UniFrac PCoA score plots (Figure 1(a,b)), suggesting that the microbiota structure of the maternal saliva samples was obviously different from that of the other sample types. On the weighted score plot, symbols representing the amniotic fluid, vaginal fluid, and meconium samples located closely, while symbols representing the maternal feces samples formed a distinct cluster (Figure 1a). The results of the PCoA performed based on the unweighted UniFrac distance were slightly different for the meconium and maternal feces samples. Symbols representing the amniotic fluid, vaginal fluid, and maternal feces samples clustered at the lower left quadrant, while symbols representing the meconium samples were distributed distinctly at the upper left quadrant (Figure 1b). Moreover, the meconium samples showed a low intragroup Bray–Curtis dissimilarity (0.6840; contrasting to 0.8214–0.9283 for the maternal samples; Figure 1(c). The intergroup distance between the amniotic fluid and vaginal fluid samples was the smallest (0.8510 versus 0.9743–0.9989 between other groups; Figure 1(c)).

### Sample type-specific OTUs

**Figure 2.**
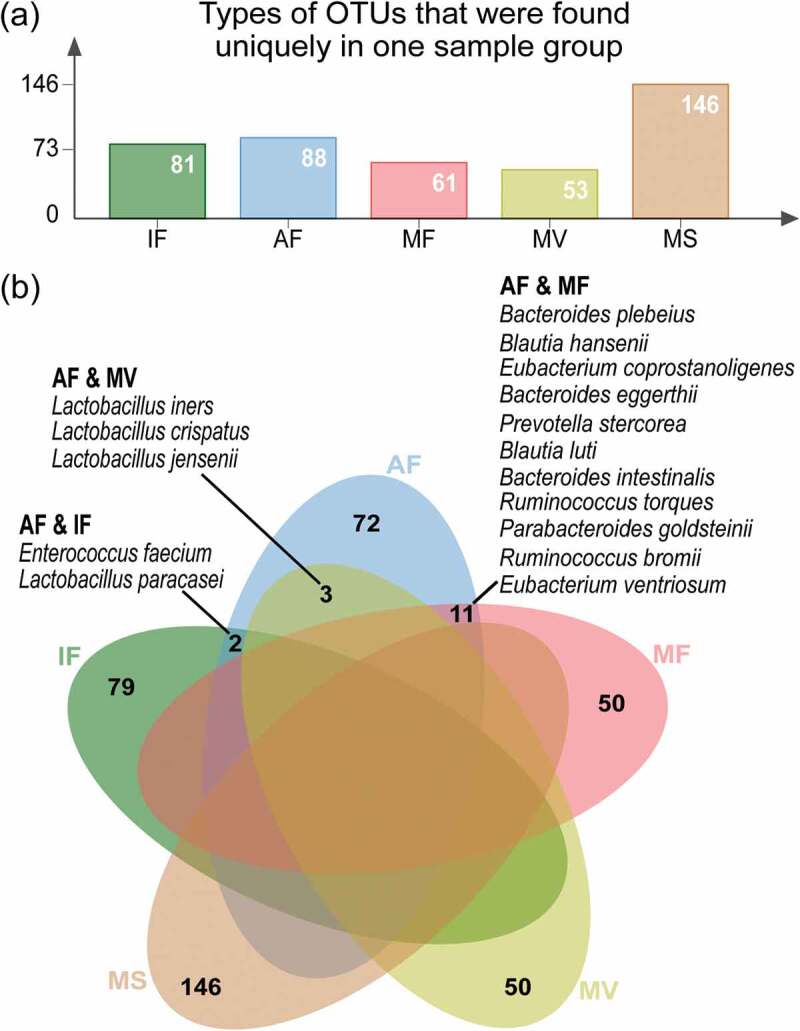
Distribution of sample type-specific operational taxonomic units (OTUs). (a) Types of OTUs that were found exclusively in one sample group. (b) Venn diagram showing common species between sample types, despite they were indeed assigned to different OTUs. The amniotic fluid-maternal feces, amniotic fluid-vaginal fluid, and amniotic fluid-meconium sample pairs shared eleven, three, and two common species, as listed (Table S2 for complete data). This analysis only included OTUs that were detected in more than two samples of the same sample group. IF: meconium; AF: amniotic fluid; MF: maternal feces; MS: maternal saliva; MV: vaginal fluid.

Sample type-specific OTUs (detected exclusively in one sample type) were identified in all sample groups (Figure 2; Table S2), supporting the existence of a sample type-specific microbiota subset in each sample group. To ensure the sample type specificity, subsequent analyses only included OTUs that were detected in more than two samples within the same sample group. The maternal saliva samples had the most types of sample-specific OTUs (147 types; 146 identified species), followed by the samples of amniotic fluid (98 types; 88 identified species), meconium (82 types; 81 identified species), maternal feces (62 types; 61 identified species), and vaginal fluid (54 types; 53 identified species) (Figure 2(a); Table S2). At the species level, the amniotic fluid-maternal feces, amniotic fluid-vaginal fluid, and amniotic fluid-meconium sample pairs shared eleven, three, and two species, respectively (Figure 2(b)).

### Prediction of the maternal origin of the meconium microbiota by SourceTracker

The SourceTracker software predicted the source of microbial communities in the input sample set.^32^ In this study, SourceTracker was used to predict the likely origin of the meconium microbiota using the maternal microbiota as potential sources.^1^ The SourceTracker analysis revealed that 8.03 ± 2.73% of the meconium OTUs matched with the maternal samples (Table 1). The 39 maternal-neonate pairs could be classified into 10 groups based on their OTU matching patterns. The meconium and amniotic fluid samples shared the highest level of common OTUs (4.12 ± 1.57%), followed by meconium-vaginal fluid sample pair (2.01 ± 1.14%) and meconium-maternal feces sample pair (1.81 ± 1.04%). The meconium and maternal saliva samples shared significantly fewer common OTUs compared with other sample pairs (0.10 ± 0.05%, *P < *.01 in all cases).

**Table 1.** Grouping of maternal-neonate pairs based on patterns of operational taxonomic units (OTUs) sharing between meconium and maternal microbiota.

	Proportion of matching OTUs (%)					
Group	AF	MV	MF	MS	Number of meconium samples in the group	Types of maternal samples that shared OTUs with the meconium sample
1	5.50	1.69	2.17	0.00	19	AF, MF, MV
2	0.00	0.00	0.00	0.00	7	None
3	0.64	0.26	0.00	0.00	4	AF, MV
4	20.03	1.45	0.00	1.06	2	AF, MS, MV
5	6.41	21.08	14.50	0.48	2	AF, MF, MS, MV
6	0.15	0.00	0.00	0.00	1	AF
7	0.00	0.00	0.00	0.31	1	MS
8	0.13	0.00	0.20	0.00	1	AF, MF
9	0.20	0.00	0.00	0.31	1	AF, MS
10	0.11	0.00	0.14	0.10	1	AF, MF, MS
Mean±SEM	4.12 ± 1.57^a^	2.01 ± 1.14^ab^	1.81 ± 1.04^b^	0.10 ± 0.05 ^c^	-	-

Different superscript letters indicate significant differences between groups (*P < *0.01 in all cases; Kruskal-Wallis test). Amniotic fluid, AF; maternal feces, MF; vaginal fluid, MV; maternal saliva, MS.

### Prediction of the maternal origin of the meconium microbiota by dyad analysis

**Figure 3.**
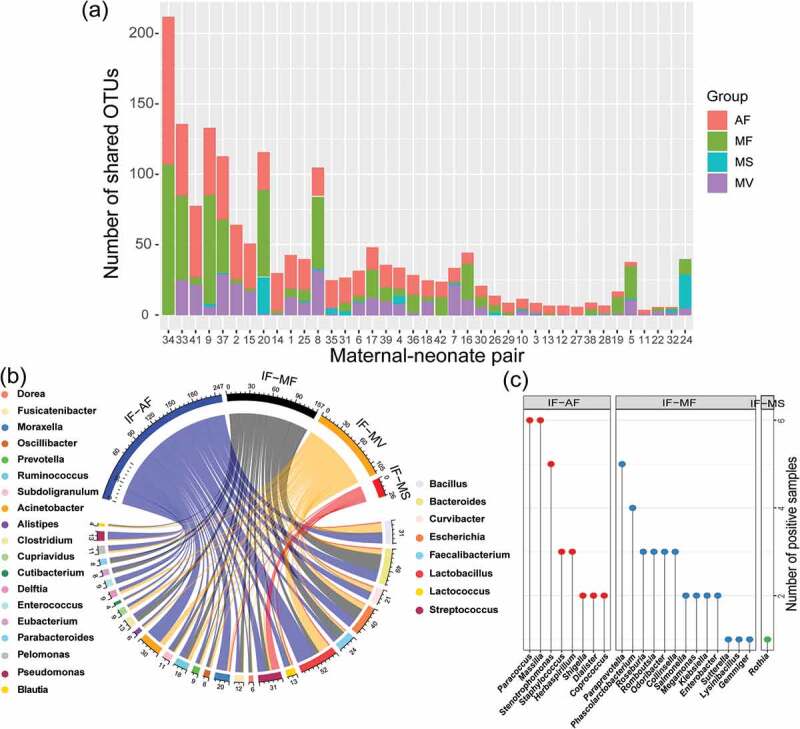
Prediction of the maternal origin of the meconium microbiota by dyad analysis. (a) Number of shared operational taxonomic units (OTUs) between the meconium microbiota and microbiota of different types of maternal samples. (b) Genus-level phylotypes of meconium OTUs that shared with two to four types of maternal samples. The upper half circle illustrates the distribution of sample sharing of these OTUs, while the lower half circle represents their genus-level phylotypes. The phylotype of each OTU shown in the upper half circle is illustrated by a line connecting to the lower half circle. Each assigned genus is illustrated by a different color, and the number written next to the lower circle represents the number of OTUs of the respective genus. The eight meconium genera listed on the right side were common to all four maternal sample types, while the 19 meconium genera listed on the left side were common to two or three types of maternal samples. (c) Meconium genera that were common to only one type of maternal sample. IF: meconium; AF: amniotic fluid; MF: maternal feces; MS: maternal saliva; MV: vaginal fluid.

To further track the maternal source of the meconium OTUs, dyad analysis was performed by matching the OTU dataset of the meconium microbiota with that of each type of maternal sample (Figure 3). Consistent with the results generated by the SourceTracker software, the amniotic fluid microbiota shared the highest number of OTU types with the meconium microbiota (18.59 ± 3.22), followed by the microbiota of maternal feces (15.20 ± 3.90), vaginal fluid (7.72 ± 1.45), and maternal saliva (2.03 ± 0.89) (Figure 3(a)). Eight meconium genera (*Bacillus, Bacteroides, Curvibacter, Escherichia, Faecalibacterium, Lactobacillus, Lactococcus, Streptococcus*) were commonly shared across all four types of maternal samples, while 19 meconium genera were in common with two or three types of maternal samples, respectively (Figure 3(b)). Eight (shared by meconium and amniotic fluid), 13 (shared by meconium and maternal feces), and one (shared by meconium and maternal saliva) common genera were identified between the microbiota of meconium and only one maternal sample type (Figure 3(c); Table S3). The meconium microbiota shared sequences representing 48, 45, 28, and 9 bacterial species with the microbiota of amniotic fluid, maternal feces, vaginal fluid, and maternal saliva, respectively (Table S4).

### The meconium microbiota of neonates delivered via cesarean section and vaginal birth

The difference in the meconium microbiota structure between neonates delivered by cesarean section and vaginal birth was assessed by PCoA and Bray–Curtis dissimilarity distance. Symbols representing the meconium microbiota of the neonates born by cesarean section and vaginal delivery did not form distinct clustering pattern on the weighted and unweighted UniFrac distance PCoA score plots (Fig. S3a; S3b), suggesting no obvious difference in the meconium microbiota structure between the two groups of neonates. Consistently, the Bray–Curtis dissimilarity of the meconium microbiota of neonates delivered by cesarean section and vaginal birth exhibited no significant difference (*P > *.05; Fig. S3c). However, the meconium samples of six vaginally delivered neonates (IF8, IF9, IF19, IF20, IF30, and IF34; PCoA1 > 0.2 and <-0.2 on the weighted and unweighted score plots, respectively) showed obvious deviation from other samples. The meconium microbiota of these six samples had significantly more *Escherichia fergusonii* (62.01% versus 3.22% in other samples; Fig. S3d) and significantly less *Bacillus cereus, Bacillus flexus, Bacillus safensis, Lactococcus piscium, Pseudomonas fragi, Oceanobacillus profundus*, and *Bacillus pumilus* (0.01%-0.39% versus 1.2%-27.21% in other samples; Fig. S3d).

### The amniotic fluid microbiota of neonates delivered via cesarean section and vaginal birth

Nonmetric multidimensional scaling (NMDS) analysis and Adonis test were performed to evaluate the difference in the amniotic fluid microbiota between neonates delivered by cesarean section and vaginal birth. Symbols representing the amniotic fluid microbiota of the neonates born by cesarean section and vaginal delivery did not form distinct clustering pattern on the NMDS score plot, suggesting no obvious difference in the structure of the amniotic fluid microbiota between the two groups of neonates. The Adonis test also found non-significant difference between the two groups (*p* = .06; Fig. S4).

## Discussion

To explore the seeding of the meconium microbiota of neonates, full-length 16S rRNA-based microbiota profiles of the meconium and maternal samples of 39 maternal-neonate pairs were analyzed. Five types of samples, including maternal feces, vaginal fluid, maternal saliva, and two low microbial biomass samples (meconium and amniotic fluid), were analyzed.^16,33^ There was no doubt that the former three sample types contained specific microbiota.^34^ The existence of microbiota in meconium and amniotic fluid was disputable due to detection methodologies and underlying contamination issues. Most previous studies were limited by employing methods that produced short amplicons. The PacBio SMRT platform could generate high-fidelity long reads when used together with the CCS strategy.^31^ Thus, this study took advantage of the PacBio SMRT platform to improve the taxonomic sensitivity and specificity of microbiota profiling as well as to decrease the risk of false positives.^16,35^

To minimize the chance of contaminating the low microbial biomass samples, both the amniotic fluid and meconium samples used in this work were collected aseptically within the first couple of hours of birth at the labor ward. The amniotic fluid samples were carefully collected from the mouths of the neonates as soon as they were born. To ensure that the amniotic fluid samples obtained from the vaginally delivered neonates were not contaminated by secondary vaginal microbial contamination during passage through the birth canal, the amniotic fluid samples were aspirated from the back of throat of the neonates. The fact that no significant structural difference was found in the amniotic fluid microbiota between neonates delivered by cesarean section and vaginal birth supported that there was no obvious secondary microbial vaginal contamination. Differing from some previous studies that collected neonates’ fecal samples after several days of birth, this work analyzed the first-pass meconium samples, which represented the fetal gut contents at birth without environmental influences like diet.^36^ Some reports found that the bacterial signatures of the microbiota of amniotic fluid and placenta were indistinguishable from the negative controls;^25,26,37^ however, such conclusions were often drawn from analyzing a relatively small sample size. The small sample size might reduce the chance of amplifying scarce DNA in low microbial biomass samples, thus lowering the detection sensitivity and resolution.

Our work detected microbiota in all five sample types; and the microbiota of each group exhibited distinct features, as evidenced by significant differences in both alpha- and beta-diversity. Moreover, sample group-specific OTUs occurring exclusively in one sample type were identified in all sample groups. Indeed many sample group-specific OTUs were detected in multiple samples within the same group. For example, the most frequently detected OTUs in the whole dataset were denovo61553, denovo210693, denovo206472, denovo229606, denovo23827, and denovo190200 of the meconium microbiota, with each of them detected in more than 30 samples. The top four maternal-saliva-specific OTUs (each detected in >20 samples) were denovo227715, denovo105413, denovo84664, and denovo178858. Despite the high detection rates of these OTUs, they were found exclusively in the meconium and maternal saliva microbiota, respectively, suggesting high sample specificity. The species-level taxonomic assignment revealed some common species between the sample pairs of amniotic fluid-maternal feces, amniotic fluid-vaginal fluid, and amniotic fluid-meconium. The fact that they were assigned to different OTUs (cutoff at 98.65% dissimilarity) reflected their different origins, albeit grouped under the same species. Although some detected species were associated with multiple human body sites, many of them were indeed highly body site-specific. For example, 47 of the 146 saliva-specific species in the current dataset were reported as representative oral microbes,^38^ while many other detected species (e.g., *Prevotella melaninogenica, Prevotella dentali*, and *Actinomyces oris*) were described as typical oral commensals.^39,40^ These data supported that each investigated sample type had unique microbiota signatures and a subset of body site-specific microbiota.

Furthermore, our data showed that apart from the amniotic fluid and vaginal fluid microbiota, which shared the same dominant genera (i.e., *Lactobacillus* and *Curvibacter*), other sample groups comprised specific dominant taxa in their microbiota (*Bacillus* and *Escherichia*/*Shigella* in meconium; *Bacteroides* and *Faecalibacterium* in maternal feces; *Streptococcus* and *Prevotella* in saliva). In consistent with some previous studies,^15,41,42^ most aforementioned genera were typical members of their respective sample sites except that the genus *Curvibacter* has never been reported in the amniotic fluid and vaginal fluid microbiota. Members of the *Curvibacter* genus were previously identified in atherosclerotic plaques and were associated with the oral microflora.^43^ It was shown that oral inoculation of pregnant mice with bacteria could lead to bacterial translocation into the amniotic ﬂuid, placenta, and fetus, supporting a hematogenous bacterial transmission route from the oral cavity and/or gut to the intrauterine environment.^8,44^ Moreover, oral, gut, and vaginal bacteria might translocate to the intrauterine environment under maternal gestational stress.^44^

The meconium microbiota was characterized by a high overall relative abundance of *Bacillus* sequences (59.76%; detected in 38/39 samples), contrasting to 3.53% detected by HITChip microarray in a previous study.^41^ The dominance of *Bacillus* sequences could be an indication of contamination from external environments like the linens in the labor ward and hospital disinfectants,^45,46^ even though aseptic techniques were strictly observed throughout the sampling process. On the other hand, both the meconium and amniotic fluid samples were collected on the same day under the same environmental conditions, and only few *Bacillus* sequences were detected in the amniotic fluid microbiota (0.06%; even less for the other three types of maternal samples, representing 0.00%-0.04% of sequences). While the microbiota of most meconium samples comprised numerous *Bacillus* sequences, six samples were dominated with sequences representing the species *Escherichia fergusonii* (average of 62.01%), a species previously isolated from meconium samples.^41^ These observations and the unique microbiota makeup of each sample type argued against the viewpoint of cross contamination due to experimental workflows and/or laboratory reagents. The dominance of sequences representing certain taxa could also be a result of PCR bias when the technique was applied to low microbial biomass samples, leading to preferential amplification of certain sequences in the samples and serious overestimation of these taxa. Thus, further dyad analysis was mainly performed based on identifying common OTU types shared between different samples of the same maternal-neonate pair rather than the absolute quantity or relative abundance of OTUs.

Despite the fact that the maternal saliva samples (8.11 ± 0.54) had a significantly higher Shannon diversity compared with other maternal sample types (3.80 ± 1.75 to 7.77 ± 0.91; *P* < .05), they had both a lower proportion (saliva: 0.10 ± 0.05%; other three types of maternal samples: 1.81 ± 1.04% to 4.12 ± 1.57%; Table 1) and a lower number (saliva: 2.03 ± 0.89; other three types of maternal samples: 7.72 ± 1.45 to 18.59 ± 3.22) of OTU match with the meconium microbiota compared with the other three types of maternal samples (*P* < .05). These results suggested that the patterns of OTU match were not purely attributed to chance coincidence. The significantly lower level of matching was likely due to the fact that the buccal cavity was not a body site that related directly with the pregnancy and childbirth processes. Additionally, based on the patterns of OTU match, the 39 maternal-neonate pairs could be classified into 10 groups. Nineteen maternal-neonate pairs showed OTU matches between the microbiota of meconium and three maternal samples (namely amniotic fluid, maternal feces, and vaginal fluid) and no match was found in seven maternal-neonate pairs, suggesting that the patterns of OTU match varied largely between individuals.

Many OTUs in our dataset were commonly shared between the meconium microbiota and microbiota of multiple maternal samples, suggesting that the meconium microbiota was likely originated from multiple maternal sites, which was consistent with the findings of Ferretti et al.^11^ Nevertheless, some genera were only shared between the meconium microbiota and the microbiota of one maternal sample type. For example, eight genera were exclusively shared between the meconium and amniotic fluid microbiota, while 13 genera were shared only between the meconium and maternal fecal microbiota. Most of these genera were previously detected in the meconium microbiota, including *Massilia, Stenotrophomonas, Staphylococcus, Shigella, Dialister, Coprococcus, Phascolarctobacterium, Roseburia, Collinessa, Salmonella, Klebsella*, and *Enterobacter*.^34^ Moreover, the genera *Massilia, Staphylococcus*, and *Shigella* were previously reported in both meconium and amniotic fluid microbiota, while *Roseburia, Collinsella, Salmonella*, and *Enterobacter* were reported in both meconium and maternal fecal microbiota.^34^ No OTU was shared exclusively between the microbiota detected in the meconium and vaginal fluid because all these common taxa overlapped with those shared between the meconium and amniotic fluid microbiota. These included members of seven genera (*Acinetobacter, Cupriavidus, Cutibacterium, Delftia, Pelomonas, Lactobacillus*, and *Pseudomonas*), all of which except *Cutibacterium* were previously detected in the microbiota of meconium and vaginal fluid/amniotic fluid.^34^ The vaginal fluid samples were collected from the subjects soon before their admission to the delivery ward and rupture of membranes, and the amniotic fluid samples were aspirated from the back of the throat of the neonates. Therefore, the amniotic fluid and vaginal fluid samples had low chance of cross-contamination during sample collection. The close resemblance of the OTU sharing patterns between the amniotic fluid-meconium and vaginal fluid-meconium sample pairs was more likely resulted from the ascending translocation of vaginal bacteria to the amniotic cavity during pregnancy.^34,47^ Moreover, the fetus swallowed large quantities of amniotic fluid during the later stages of pregnancy, which could lead to a higher similarity between the amniotic fluid and meconium microbiota.^48^

Hitherto, Stinson et al. have been the only published study that applied PacBio sequencing to describe the microbiota of meconium and amniotic fluid samples of maternal-neonate pairs.^16^ Large differences were observed in the microbiota composition of the meconium and amniotic fluid samples between this study and Stinson et al. The discrepant results could be due to both an additional PCR decontamination step (using a double-strand specific DNase) in Stinson et al. and natural inter-ethnic/-individual variations in the microbiota composition. The incorporation of such decontamination step could minimize the risk of contamination from reagent kits, but meanwhile increased the chance of accidental removal of template DNA, compromising the microbiota diversity. Nevertheless, Stinson et al. and the current study identified 15 common meconium-associated species and 25 common amniotic fluid-associated species (Table S5), suggesting commonalities in the microbiota composition of samples collected from independent sources. Stinson et al. found that most meconium microbiota contained abundant *Pelomonas puraquae* sequences, and there was an intense negative correlation between the number of *Pelomonas puraquae* reads and the propionate level in the meconium samples, strongly suggesting that this species had true biological significance.^16^ In the current dataset, *Pelomonas puraquae* sequences were detected in only five amniotic fluid samples, but *Pelomonas saccharophila* sequences were found in 13 meconium, 28 amniotic fluid, 18 vaginal fluid, three maternal feces, and one saliva samples (Table S6). Ten of the 13 *Pelomonas saccharophila* sequence-positive meconium samples also had *Pelomonas saccharophila* sequences in their amniotic fluid and/or vaginal fluid counterparts. Conversely, even *Pelomonas saccharophila* sequences were detected in three maternal feces and one saliva samples, their meconium counterparts were *Pelomonas saccharophila* sequence-negative. Our data supported that *Pelomonas* was a part of the meconium microbiota, and this species might be seeded from the amniotic fluid and/or vaginal tract microbiota.

This work suffered from some limitations. There might be some technical issues like batch effect or PCR bias, particularly when analyzing low microbial biomass samples. For example, the observation of non-significant difference in the structure of the meconium microbiota between neonates born by cesarean section and vaginal birth (except six vaginal cases) could have been a result of batch effect or other underlying technical issues. Another significant limitation was that contaminant and extraction blank controls were not included; thus, this work could not completely rule out the chance of contamination acquired during the experimental workflow.

In conclusion, the facts that some meconium OTUs were also detected in multiple maternal samples and that the meconium microbiota shared more features with the amniotic ﬂuid microbiota than with microbiota of other maternal samples, suggesting that the meconium microbiota was likely seeded from multiple maternal sites and that the amniotic sac contributed more significantly than other investigated maternal body sites in this process.

## Materials and methods

### Human subjects

Thirty-nine healthy pregnant women (mean age = 28.64 ± 4.13; median age = 28; age range = 22–40), who visited the Department of Gynecology and Obstetrics of the Affiliated Hospital of Inner Mongolia Medical University, were recruited. One hundred ninety-two samples were collected from 39 maternal-neonate pairs, including first-pass meconium (39), maternal feces (39), amniotic fluid (39), vaginal fluid (39), and maternal saliva (36). All neonates were born at full-term (eight delivered by elective cesarean section and 31 delivered vaginally). No subject received any antibiotics during pregnancy. This work was approved by the Ethical Committee of the Affiliated Hospital of Inner Mongolia Medical University, Inner Mongolia, China. Written informed consent was obtained from all participated mothers prior to the study.

### Sample collection

All samples were collected in sterile containers having an equal volume of sterile cryoprotectant. First-pass meconium samples (~15 g) were collected on sterilized diapers by the delivery doctors within the first few hours of birth at the labor ward. Samples of maternal feces (~15 g) and saliva (~4 mL) were collected by the mothers within 24 hours before giving birth. Maternal saliva samples were collected with a straw one-hour after meal, and the collected saliva was transferred to a sterile cryotube containing the cryoprotectant. Vaginal fluid samples (~4 mL) were collected from the participants with sterilized cotton swabs by the responsible medical doctors prior to admission to the delivery ward and rupture of membranes. Amniotic fluid samples (~2 mL) were the aspirates collected from the back of the throat of the neonates to minimize the chance of introducing secondary vaginal microbial contamination to the amniotic fluid samples of vaginally delivered neonates. The amniotic fluid samples were aspirated using sterile plastic pipettes by the delivery doctors immediately after birth prior to cord clamping. All samples were stored temporarily in an ice box and were transported promptly to the laboratory at 4°C. Thereafter, the samples were stored at −80°C until further processing.

### Metagenomic DNA extraction

All molecular biology works were performed meticulously in a strictly controlled, separate, and sterile workplace. The metagenomic DNA of samples was extracted by the QIAamp Fast DNA Stool Mini Kit (Qiagen GmbH, Hilden, Germany) following the instructions of the manufacturer. Agarose gel electrophoresis and a Nanodrop spectrophotometer were used to ensure the quality of the extracted DNA. Extracted DNA was stored at −20°C until use.

### 16S rRNA amplification and SMRT sequencing

Full-length 16S rRNA was amplified by PCR with universal primers (27 F, AGAGTTTGATCMTGGCTCAG; 1492 R, ACCTTGTTACGACTT).^49^ The 5ʹ primer ends were tagged with paired 16-nt symmetric barcodes. The thermocycling program was: 1 cycle of 95°C for 5 min, followed by 28 cycles of 95°C for 1 min, annealing at 58°C for 1 min, and 72°C for 2 min, followed by 1 cycle of 72°C for 10 min. The Agilent DNA 1000 Kit and an Agilent 2100 Bioanalyser (Agilent Technologies) were used for amplicon quantification. Amplicons (2 μg/sample) were used for constructing DNA libraries with the Pacific Biosciences Template Prep Kit 2.0. Sequencing was performed in the CCS mode on a PacBio RS II instrument with the P6/C4 chemistry.^29,31^

### Sequence analyses

Raw data were processed by the protocol RS_ReadsOfinsert.1 (available under the SMRT Portal, version 2.7). The restrictive filter criteria were: (i) minimum full passes of up to 5; (ii) minimum predicted accuracy of 99; (iii) minimum insert read length of 1400; and (iv) maximum insert read length of 1800. Firstly, all reads were classified based on sample-specific primer barcodes. Barcode sequences were removed before extracting high-quality sequences by the Quantitative Insights Into Microbial Ecology package (QIIME; version 1.7). Then, the most abundant sequence of each cluster was selected as a representative to be aligned by PyNAST (100% clustering of sequence identity)^50^ and UCLUST.^51^ Afterward, the unique sequences were assigned to OTUs by UCLUST (cutoff at 98.65%).^52^ Chimeric OTU sequences were screened and removed by ChimeraSlayer.^53^ The remaining OTUs were taxonomically assigned by the Ribosomal Database Project (RDP) II and Greengenes (version 13_8) databases (minimum bootstrap threshold of 80%).^54,55^

Alpha- and beta-diversity were calculated from the *de novo* taxonomic tree constructed by the representative chimera-checked OTU dataset using FastTree.^56^ To assess the sequencing depth and biodiversity richness, rarefaction curves and rank–abundance curves of all samples were constructed. The Shannon diversity index was calculated. The potential maternal origin of the meconium microbiota was predicted by SourceTracker (version 0.9.5).^32^ Then, the OTU sharing patterns between the microbiota of the meconium and maternal counterpart samples of each maternal-neonate pair were analyzed. The Venn diagram was created by jvenn.^57^

### Statistical analyses

Statistical analyses were performed mainly with R packages (http://www.r-project.org/). Data were expressed in mean±SD unless otherwise stated. Principal coordinate analysis was performed based on the weighted and unweighted UniFrac distances to evaluate the structural difference in the microbiota between different sample groups.^58^ The Bray–Curtis dissimilarity matrix, NMDS, and Adonis test were performed by the Vegan package in R. Significant differences between groups were evaluated by Mann-Whitney test or Kruskal–Wallis test at a confidence level of 0.05.

## Supplementary Material

Supplemental Material

Supplemental Material

## Data Availability

Raw sequence data were deposited to the MG-RAST metagenomics database (Project number mgp79730).
