# Supplementary material for: The meconium microbiota shares more features with the amniotic fluid microbiota than the maternal fecal and vaginal microbiota
Source: Gut Microbes. 2020 Aug 2;12(1):1794266. doi: 10.1080/19490976.2020.1794266 (PMC7524391; doi:10.1080/19490976.2020.1794266)
Supplement: Supplemental Material [file KGMI_A_1794266_SM9622.doc]

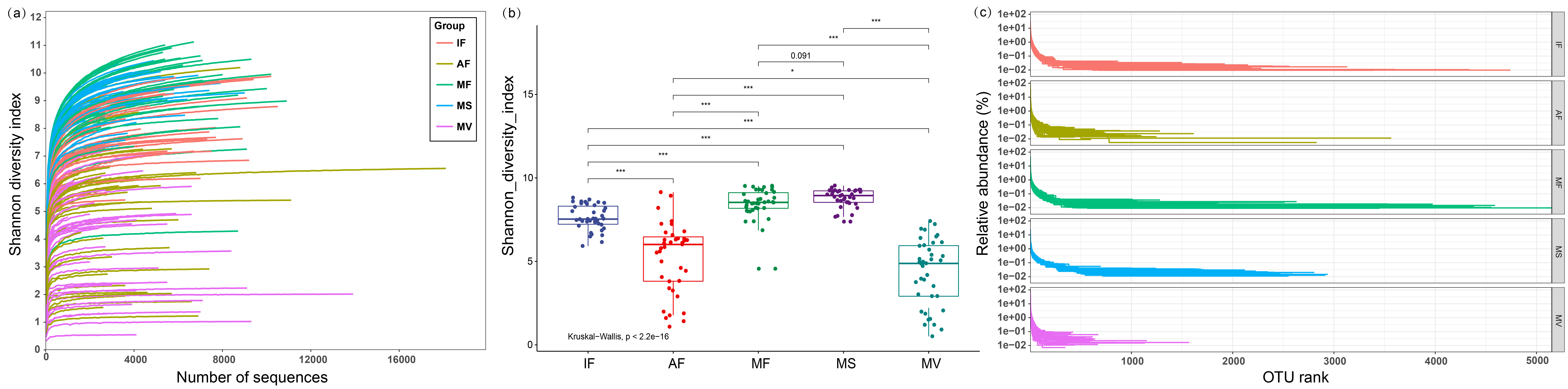


Figure S1 Alpha diversity. Rarefaction curves of all samples (a); box-plots showing the Shannon diversity index of five different sample groups (b); rank-abundance curves of all samples (c). 'IF', 'AF', 'MF', 'MS', and 'MV' represent meconium, amniotic fluid, maternal feces, saliva, and vaginal fluid, respectively. The single asterisk and triple asterisk represent statistical significance at *P* < 0.05 and p < 0.001, respectively (Mann-Whitney test).





Figure S2 Stacked bar charts and boxplots showing microbiota compositions of five types of samples at different taxonomic levels. IF: meconium; AF: amniotic fluid; MF: mother feces; MS: mother saliva; MV: vaginal fluid. Phylum (a, b); genus (c, d); species (e, f).


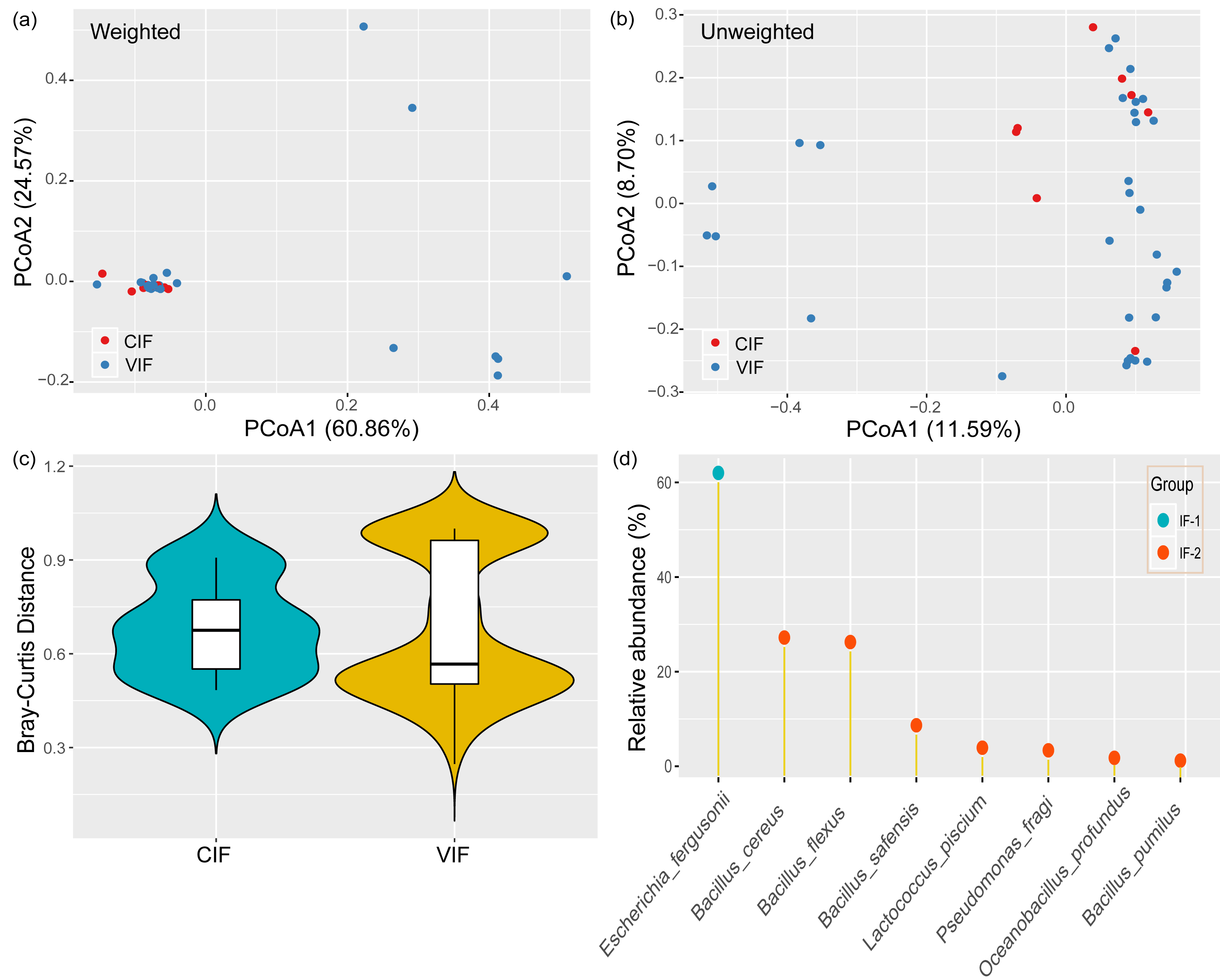


Figure S3 Differences between the meconium microbiota communities of neonates delivered by cesarean section (CIF) and vaginally (VIF). Scores plots of principal coordinates analysis of weighted and unweighted UniFrac distances (a, b); violin plot showing Bray-Curtis distances of the two groups (c); relative abundances of significant differential abundant species in subgroup 1 (IF-1) and subgroup 2 (IF-2). Subgroup 1 comprised six deviated samples (IF8, IF9, IF19, IF20, IF30, and IF34; corresponding to samples with PCoA1 >0.2 on the weighted score plot and <-0.2 on the unweighted score plot). Subgroup 2 comprised all other samples (d).


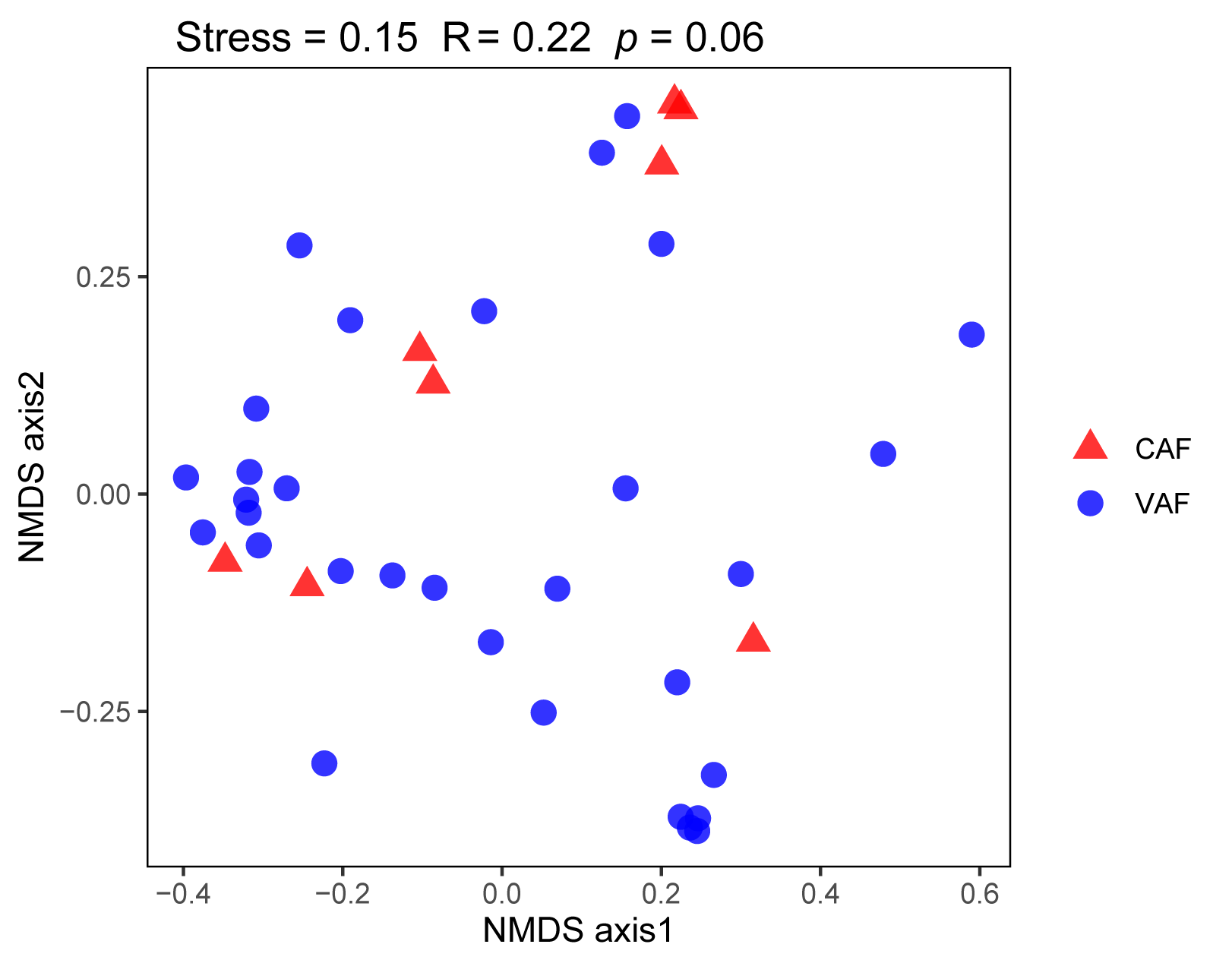


Figure S4 Nonmetric multidimensional scaling (NMDS) analysis of the amniotic fluid microbiota of neonates delivered by cesarean section (CAF) and vaginally (VAF). Adonis test was performed to evaluate the difference in the amniotic fluid microbiota between the two groups.
